# Effects of Steel Fiber and Specimen Geometric Dimensions on the Mechanical Properties of Ultra-High-Performance Concrete

**DOI:** 10.3390/ma15093027

**Published:** 2022-04-21

**Authors:** Haozhen Fang, Mingen Gu, Shufeng Zhang, Haibo Jiang, Zhuangcheng Fang, Jiaxin Hu

**Affiliations:** 1 School of Civil and Transportation Engineering, Guangdong University of Technology, Guangzhou 510006, China; 2112009031@mail2.gdut.edu.cn (H.F.); 2111509033@mail2.gdut.edu.cn (Z.F.); 2 Guangzhou Communication Design Institute Co., Ltd., Guangzhou 511400, China; gmingen@163.com (M.G.); zsf13570481603@163.com (S.Z.); 3 Department of Civil and Environmental Engineering, Michigan State University, East Lansing, MI 48823, USA; hujiaxi1@msu.edu

**Keywords:** ultra-high-performance concrete, steel fiber, specimen geometric dimension effect, mechanical properties, constitutive law

## Abstract

Ultra-high-performance concrete (UHPC) is an advanced concrete with superior mechanical strength, ductility and durability properties. However, the influence of steel fiber on its constitutive laws and the specimen geometric dimension effect on its strength had not been paid enough attention. To investigate the effect of steel fibers on the properties of UHPC, specimens with different fiber volume contents and fiber types were tested. Meanwhile, the mechanical properties of UHPC at different ages from 3 days to 28 days were conducted. Moreover, specimens with various geometric dimensions were also prepared to study the effect of specimen geometric dimensions (dog-bone-shaped, prism and cylinder specimens) on the properties of UHPC. The results indicated that elastic modulus, tensile peak stress and the corresponding strain increased as the fiber volume content and curing age increased. Specimens with hooked-end fibers exhibited better tensile performance than those with straight fibers. Furthermore, different geometric dimensions of specimens significantly influenced the tensile properties of UHPC. Based on the experimental results, conversion factors were suggested for the transformation of strength obtained from specimens with different geometric dimensions to reference specimens. In addition, both compressive and tensile constitutive laws were proposed to generate the stress–strain relationship of UHPC.

## 1. Introduction

As a newfangled cement-based composite material, ultra-high-performance concrete (UHPC) had been widely used in structural engineering in the past decades [1,2,3,4]. Owing to the optimized gradation of granular components and high range of water reducer, outstanding mechanical properties could be achieved with a low water-to-binder ratio of less than 0.25 [5,6]. Furthermore, the lack of coarse aggregates and the optimization of granular mixture offered the matrix an enhanced microstructure [7,8]. Accordingly, UHPC exhibited superior compressive strength (over 120 MPa) and sustained post-cracking tensile strength (over 5 MPa) [9,10,11]. Moreover, owing to the effective bonding between matrix and discontinuous fiber, steel fiber played a critical role in the stress–strain relationship of UHPC [12]. However, the mechanical properties of UHPC obtained from specimens with different geometries and dimensions varied significantly [13]. Few studies focused on the impact of steel fiber on the constitutive laws of UHPC, as well as the specimen geometric dimension effect on the mechanical properties of UHPC.

As an important index for structural design, the mechanical properties of UHPC were affected by the fiber characteristics. The flowability of fresh UHPC mixtures decreased as fiber volume content and length increased [12,14]. In addition, ductile failure modes were observed in UHPC owing to the random distributed steel fiber. In addition, brittle failure could be changed into ductile failure due to the existence of steel fibers [15]. Furthermore, increasing the steel fiber length and volume contents could also improve the performance of UHPC significantly. However, lower compressive and elastic modulus were obtained when the steel fiber length reached 13 mm and with steel fiber volume content approaching 4% [9]. Moreover, the capacities of UHPC with different types of fibers were various due to the fiber geometries. The use of hooked-end or twisted steel fibers always showed higher tensile strength than the UHPC with straight fibers [16].

Although, the existing UHPC constitutive laws could barely accurately describe the stress–strain relationship accurately owing to the complex mechanism resulting from the random distributed steel fiber. As the only code that took the steel fiber distribution into account, fiber orientation factor *K* was introduced to express the tensile/compressive constitutive laws by the AFGC recommendation [17]. Moreover, linear and exponential models were proposed in previous studies to predict the stress–strain relationship of UHPC [10,18,19,20,21,22,23]. A linear equation related to the elastic modulus and the reduction factor of stress was defined by Graybeal to describe the ascending portion of the compressive stress–strain relationship [18]. Nielsen proposed an exponential function to describe the whole stress–strain curve in compression [19]. Additionally, piecewise functions of constitutive laws were also adopted to simulate the behavior of UHPC in other studies [10,18,19,20]. Moreover, the volume content and lengths of steel fiber were taken into account by Wu et al. [10]. Based on the tensile test of prisms, exponential functions were also used to describe the curve of the tensile stress–strain relationship [22]. Accordingly, multiple linear functions were also proposed to consider the stress–strain behavior in tensile stress–strain curves [21,23]. However, most of the empirical formulas mentioned above were derived from linear regression analysis, which could not fully consider the effect of steel fibers.

Additionally, the properties of UHPC were also sensitive to the geometries and dimensions of specimens [16]. As an important factor affecting the fiber bridging effect, the fiber distribution characteristic was significantly influenced by the geometries and dimensions of UHPC specimens [24]. Furthermore, the loading procedures appropriate for different specimen geometries also led to variation in UHPC mechanical properties. Therefore, the geometric dimension effect revealed the mechanical properties of UHPC changing with different geometries and dimensions of specimens both in compression and tension. In current design codes, cube and cylinder specimens were recommended for determining the compressive strength of UHPC [17,25,26,27], whereas the tensile strength could be obtained from dog-bone shaped (direct tension), prism (bending test), and cube or cylinder (splitting test) specimens [26,27,28,29]. The research about size effect of HSC suggested the static compressive strength increased with the decrease in aspect ratio of the specimen [30]. In addition, the strength was independent of the cube dimension for fiber-reinforced concrete with strength over 140 MPa [13]. To take size and geometric effects into account, conversion coefficients should be considered to transform the strength of specimens with different geometric dimensions both in compression and tension [13,15]. For specimens with a given geometry, the conversion coefficients would decrease with the increase in compressive strength [13,31]. In addition, the steel fibers were distributed randomly with the increase in specimen dimensions, leading to the degradation of tensile strength [24]. However, the abovementioned studies were mainly concentrated on the size effect rather than the geometric dimension effect of UHPC.

This study aimed to investigate the mechanical properties of UHPC, as well as the geometric dimension effect. Four types of steel fibers were adopted to evaluate the effects of fiber volume content, fiber type, and curing age on the mechanical properties of UHPC. The workability, failure mode, stress–strain relationship, elastic modulus, Poisson’s ratio, peak stress and corresponding strain were then studied. Moreover, the surface strain was analyzed by digital image correlation (DIC) technology. The compressive stress of cube and cylinder specimens with different dimensions and tensile stress of dog-bone shape (direct tensile test), prism (axial tensile test), prism (four-point bending test), and cylinder (splitting test) specimens were compared. Moreover, conversion coefficients of compressive strength for cylinder and cube specimens were proposed, and the conversion coefficients of tensile strength among specimens with different geometric dimensions were also presented. Based on the experimental results and previous research, constitutive laws considering fiber volume content, fiber geometry and fiber length were proposed.

## 2. Experimental Program

### 2.1. Raw Materials

The binder materials of the UHPC matrix included P·II 52.5R Portland cement [32], silica fume, silica sand, and Nano-CaCO_3_ powder. Silica sand was used as fine aggregate with a specific surface area of 2.62 g/cm^3^ and a fineness modulus of 1.58. Nano-CaCO_3_ powder with fineness of 100 nm was also adopted in the mixture as an interfiller to enhance microstructure. A high range of water reducer with an actual water reduction of about 34% was utilized to improve the workability of fresh UHPC mixtures.

**Figure 1:**
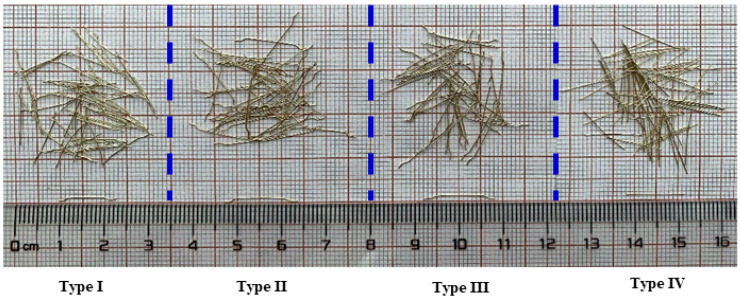
Four types of steel fibers.

Additionally, four types of brass-coated steel fibers were incorporated into the matrix in this study, as listed in Table 1 and shown in Figure 1. Three types of hooked-end steel fibers with various aspect ratios (*l_f_*/*d_f_*) of (59.09 for **Type I**, 72.73 for **Type II** and 64.00 for **Type III**), and one type of straight fiber (**Type IV**) with a length of 13 mm, and diameter of 0.20 mm were used.

### 2.2. Mix Procedure and Specimen Preparation

#### 2.2.1. Mix Procedure

**Figure 2:**
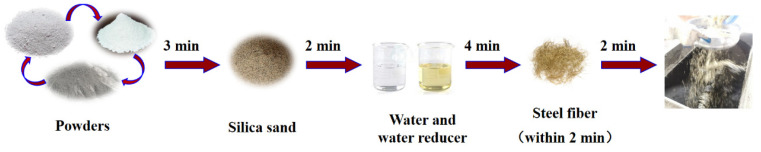
Fabrication of UHPC mixture.

The UHPC mixture proportions in this investigation are summarized in Table 2. Specially, fiber volume content was neglected in the mix proportions. As illustrated in Figure 2, the same mix procedure was adopted for all mixtures. The dry powders (i.e., cement, silica fume and Nano-CaCO_3_) and silica sand were premixed before adding the water and water reducer. The materials were mixed for another 4 min to give the mixture enough fluidity. Afterwards, steel fiber was added by passing a sieve to ensure proper dispersion. Specially, the unit weight and flowability recommended by ASTM C1437-15 for fresh concrete were also tested [33]. A layer of fresh concrete about 25 mm in thickness was place in the mold at the center of the flow table and tamped. The table was immediately drop 25 times in 15 s once lifting the mold away [33]. All specimens were demolded at about 24 h after casting and then cured in a moist condition for the next 7 days. Finally, all specimens were placed outside the laboratory for natural curing until the testing day (generally 28 days after casting).

#### 2.2.2. Compressive Specimen Preparation

**Figure 3:**
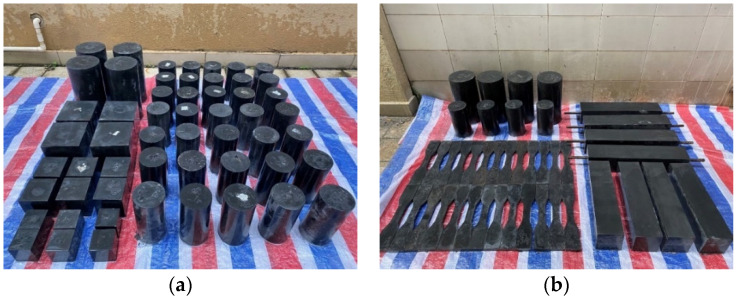

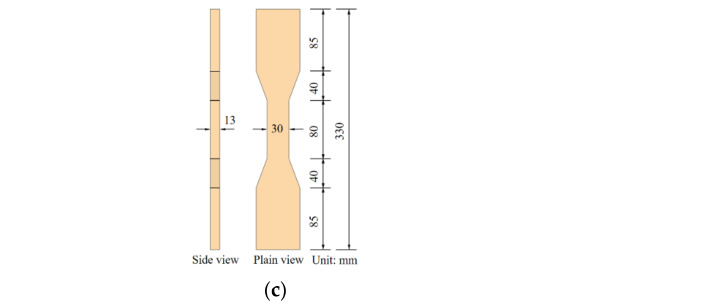
Test specimens: (**a**) compressive specimens; (**b**) tensile specimens; (**c**) dimensions of dog-bone specimens.

As shown in Figure 3a, cylinder and cube specimens with different sizes were conducted for compressive tests in the study. A number of ϕ100 × 200 mm^2^ cylinders were taken as reference specimens to obtain the properties of UHPC in compression, such as elasticity modulus, compressive strength and the stress–strain relationship. In addition, the effect of fiber volume contents was investigated by incorporating 0%, 1%, 2% and 3% of **Type I** steel fiber in the ϕ100 × 200 mm^2^ cylinders. The effect of fiber types was also investigated by incorporating **Type I**, **Type II**, **Type III** and **Type IV** steel fibers in the ϕ100 × 200 mm^2^ cylinders. Furthermore, the mechanical properties at 3 and 7 days were tested. The detailed parameters of the above compressive specimens can be obtained from Table 3. Moreover, at least three specimens were contained in each group to minimize the experimental deviations.

#### 2.2.3. Tensile Specimen Preparation

The dog-bone shaped specimens (recommended by JSCE [28]) were taken as reference specimens in the tensile test (Figure 3c). The parameters tested for tensile properties were basically the same as those for the compressive test. In order to investigate the fiber volume content and type effects on the direct tensile test, **Type I** steel fiber with different volume contents and different types of steel fibers with a volume content of 2% was adopted. In addition, dog-bone shaped specimens with different curing days were tested. Additionally, the splitting tensile test recommended by ASTM C496 [29], the four-point bending test, and the axial tensile test recommended by NF P 18-710 were also conducted, as presented in Figure 3b [27].

### 2.3. Experimental Setup

#### 2.3.1. Compressive Test

**Figure 4:**
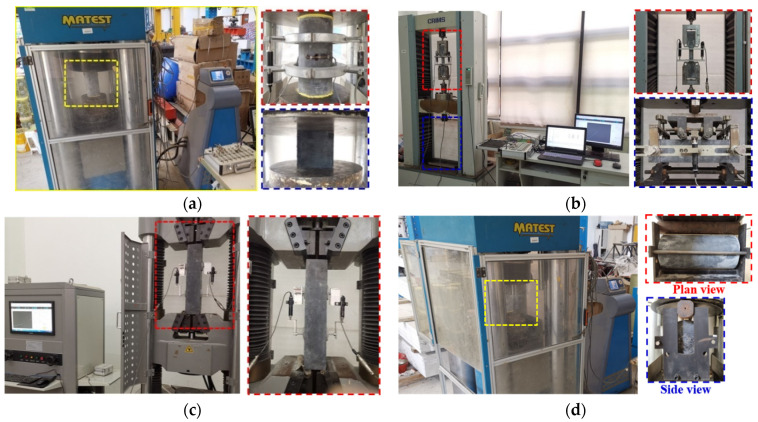
Test setup and instrumentation: (**a**) compressive test; (**b**) direct tensile and four-point bending tests; (**c**) axial tensile test; (**d**) splitting tensile test.

As shown in Figure 4a, the compressive tests were conducted using an electro–hydraulic servo machine with a maximum load carrying capacity of 4000 kN. All the cylinder specimens were coated with gypsum to minimize uneven surfaces at each end before testing. Two LVDTs were placed on the opposite sides of the cylinders by two circular rings, as recommended in ASTM C469 [29]. The loading rate applied to the cylinders specimens was set as 0.04 mm/min [34].

As for the cube specimens, only compressive strengths were measured to investigate the geometric dimension effect. All cubes were loaded at a rate of 0.5 MPa/s [13]. The test terminated when the load carrying capacity decreased to 80% of the peak strength.

#### 2.3.2. Tensile Test

The test on dog-bone shaped and prism specimens were carried out on universal testing machines (Figure 4b,c), while the splitting test was executed on the same machine as the compressive test (Figure 4d). The dimension and test frame of the dog-bone shaped specimen were according to JSCE [28], in which a loading rate of 0.5 mm/min was recommended. The four-point bending test was conducted at a rate of 0.2 mm/min with vertical deflection; LVDTs were arranged at the front and rear of the specimens. Specially, a universal testing machine with a larger displacement range was adopted for the axial tensile test owing to the longer length of T-ZL specimens. Splitting tensile tests of cylinders with two different sizes were tested according to ASTM C496 [29], with a rate of 1 kN/s.

#### 2.3.3. DIC Technology

**Figure 5:**
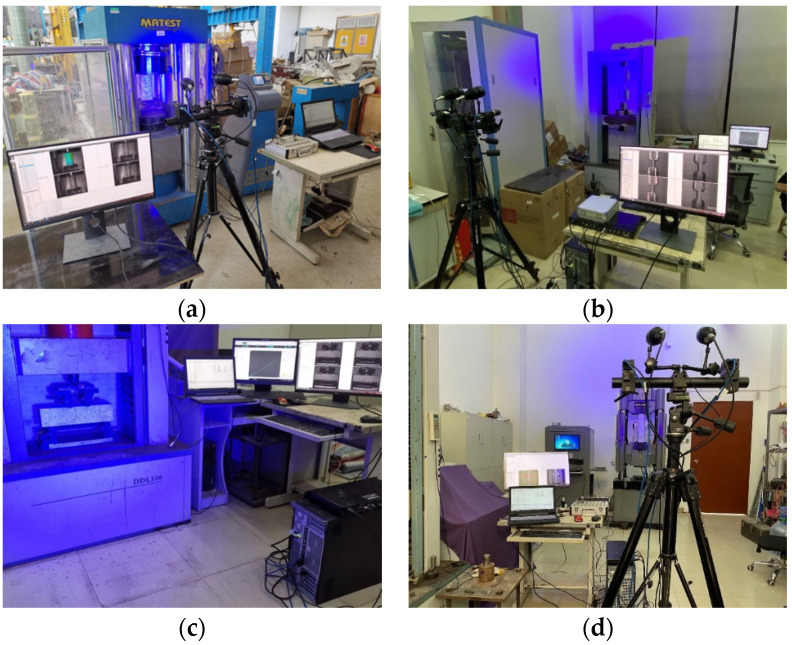
DIC testing of specimens: (**a**) compressive test; (**b**) direct tensile; (**c**) four-point bending test; (**d**) axial tensile test.

Digital Image Correlation (DIC) is a non-destructive non-contact optical monitoring technique, which could be used to analyze structural surface strains and crack development patterns [35]. Two cameras were used to take a series of grayscale digital images from two different angles aimed at the surface of the specimen. In addition, the strain and crack development patterns on the specimen surface were calculated by the 3D DIC software. In this study, speckle patterns were sprayed firstly on the surface of specimens (cylinders in compression, dog-bone-shaped specimens, and two types of prisms in four-point bending and axial tensile tests). Two high-resolution cameras which captured images every 1 s were positioned in front of the specimens. In addition, two blue lights were used to avoid the effect of frequent blinking light on the specimens (Figure 5).

## 3. Results and Discussion

### 3.1. Workability

**Figure 6:**
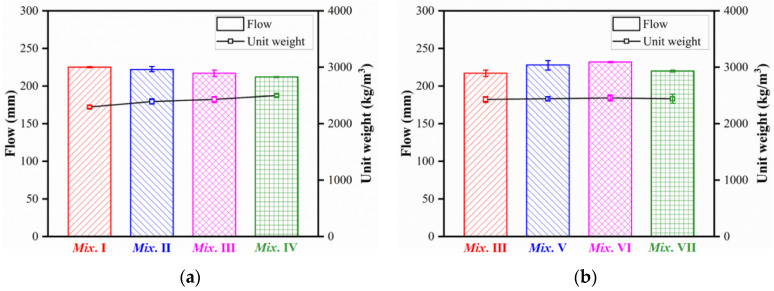
Flowability and unit weight of fresh UHPC mixtures: (**a**) effect of fiber volume contents; (**b**) effect of fiber types.

The measured slump flow and unit weight of the fresh UHPC mixtures are summarized in Table 2. Figure 6 presents the effects of steel fiber volume content and fiber type on the slump flow and unit weight of the fresh UHPC mixtures. The slump flow of the mixture without fiber was 225 mm, while UHPC with 1%, 2%, and 3% fiber volume content were 222, 217, and 212 mm, respectively. Mixtures with different types of fibers recorded slump flows ranging from 217 to 232 mm. The slump flow did not undergo a significant change with different fiber volume contents or types, which were attributed to the high aspect ratio of the fibers. Martinie et al. suggested that fiber with a high aspect ratio and low volume content (far lower than 3.2/*r*) had an inconspicuous impact on the rheological behavior of the cementitious material, with *r* the aspect ratio (*l_f_*/*d_f_*) [36]. The highest volume content and lowest ratio of 3.2/*r* were 3% and 5.2%, respectively. However, the unit weight increased when the fiber content increased. The unit weight of concrete without fiber was 2294 kg/m^3^, while the unit weight of UHPC with 1, 2, and 3% fiber contents were 2392, 2429, and 2498 kg/m^3^, respectively.

### 3.2. Failure Pattern

#### 3.2.1. Reference Compressive Specimen (ϕ100 × 200 mm^2^ Cylinder)

**Figure 7:**
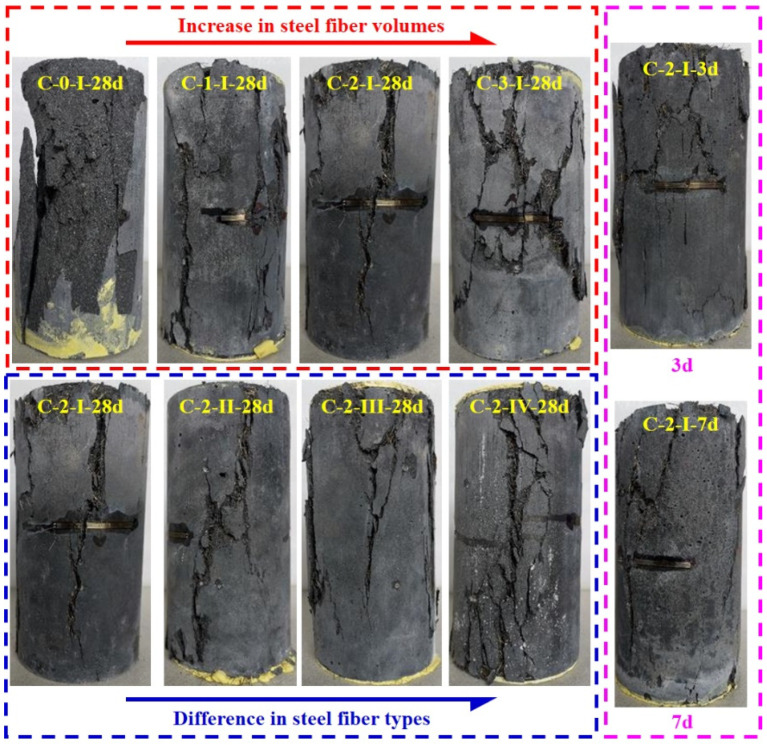
Failure patterns for the compressive test (ϕ100 × 200 mm^2^ cylinders).

Figure 7 presents the typical failure patterns of the reference compressive specimens (ϕ100 × 200 mm^2^ cylinders), in which only vertical and diagonal cracks were observed. Taking specimen C-2-I-28d as an example, specimens C-2-I-28d experienced a significant load drop after the peak stress, accompanied by concrete cracking and steel fibers pulling out.

Specimens with steel fiber volume of 0%, 1%, 2% failed mainly due to the formation of vertical cracks, while cylinders with a fiber volume of 3% failed mainly in diagonal direction. Specimens without fibers exhibited an extremely brittle failure mode, which went through an explosion with ultimate strength loss. Moreover, specimens with fiber volume of 3% presented more multiple cracks compared with specimens with low fiber volumes, which could lead to higher failure strain and higher residual strength [12]. Different types of fibers caused a slight difference in the failure pattern (with fiber volume of 2%), in which specimens with hooked-end fibers mainly exhibited vertical cracks. However, specimens with straight fibers showed more multiple cracks than the specimens with hooked-end fiber, which could be related to the weak fiber bridging effect played by the straight fibers. Additionally, both vertical and diagonal cracks were exhibited in specimens tested at the age of 3 and 7 days. Heavier concrete splitting was also observed when comparing with specimens tested in 28 days. The main reason may be the low strength of the matrix and interaction between the fiber and the matrix at an early age.

#### 3.2.2. Compressive Specimens with Different Geometric Dimensions

**Figure 8:**
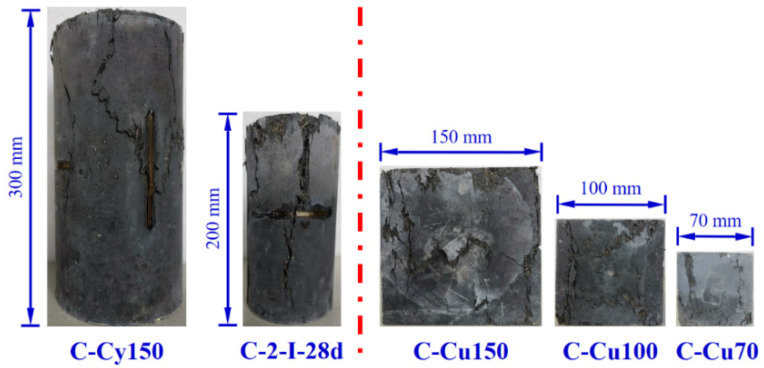
Failure patterns for compressive specimens with different geometric dimensions.

Typical failure patterns for specimens with different geometric dimensions are shown in Figure 8. Similar cracking patterns were obtained from cylinder compressive specimens with different dimensions (C-Cy150 and C-2-I-28d). However, brittle failure was prevented by fibers in ϕ150 × 300 mm^2^ cylinder specimens. As for cubes with different dimensions, spalling lateral sides were transformed into columnar cracks due to the incorporation of fibers. Coincidentally, the failure patterns did not vary much with cube size.

#### 3.2.3. Reference Tensile Specimen (Dog-Bone Shaped Specimen)

**Figure 9:**
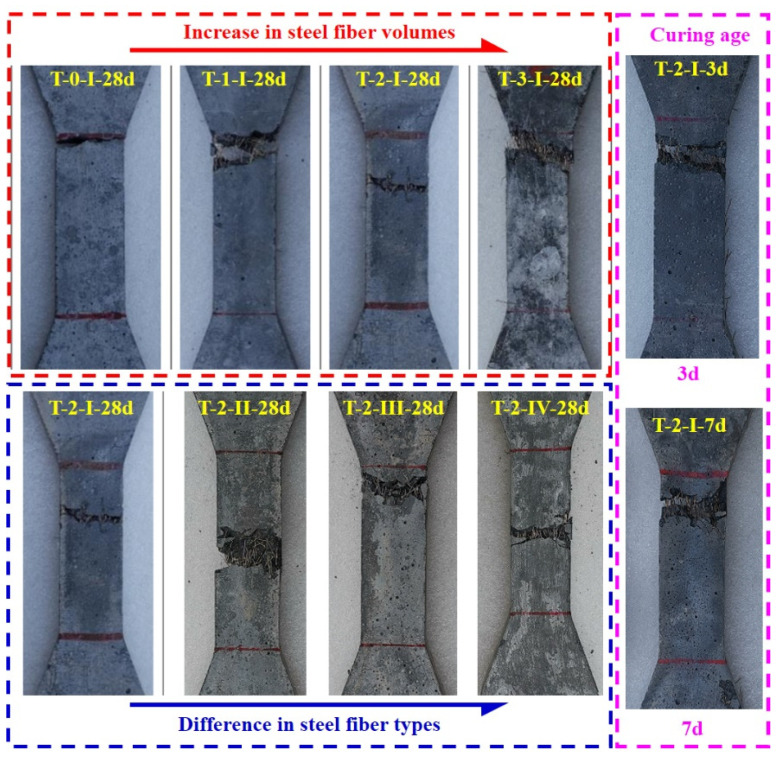
Failure patterns for the tensile test (dog-bone-shaped specimens).

Figure 9 depicts the typical failure modes of the reference tensile specimens (dog-bone shaped specimens). Specimens without steel fibers showed rapid load drop once a visible crack appeared. As for specimens with steel fibers, a single and localized crack was observed when microcracks combine to form a macrocrack at the weakest section. Afterwards, strength started to decrease after the strain–harden stage with the macrocrack width increasing until failure. However, the ultimate tensile strength increased significantly as the fiber volume increased from 0 to 3%.

#### 3.2.4. Tensile Specimens with Different Geometric Dimensions

**Figure 10:**
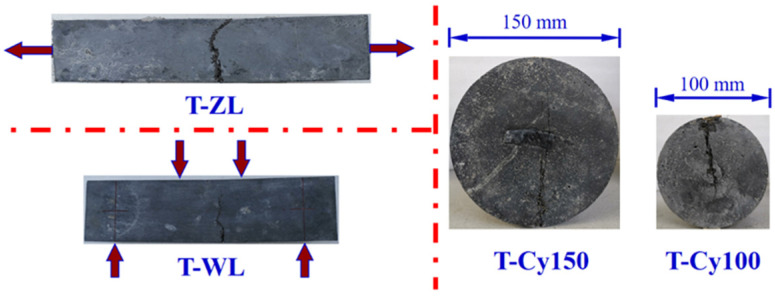
Failure modes for tensile test specimens with different geometric dimensions.

The typical failure patterns of tensile specimens with different geometric dimensions are shown in Figure 10. Due to the similar geometry and loading procedure with the reference specimens, axial tensile specimens (T-ZL) also presented a single crack. In addition, visible flexural cracks extending from the bottom to the side face were found in a four-point bending test (T-WL). As for the splitting tensile specimens with different sizes (T-Cy), specimens failed due to the local maximum strain in a single crack.

#### 3.2.5. Results of DIC

**Figure 11:**
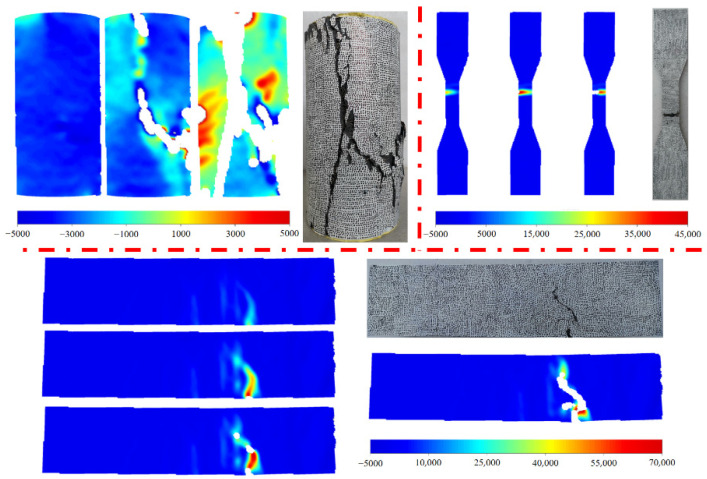
Comparison of failure modes from DIC and experiments.

The strain cloud diagrams obtained from the DIC technology were compared with the failure patterns, as shown in Figure 11. Surface strains and crack development patterns of specimens depicted that the DIC technique could capture experimental data during the test. The surface strain of axial tensile specimens was incomplete due to the impact of bright light. However, the experimental results and previous studies depicted DIC-enabled the measurement of non-uniform surface displacement due to material heterogeneity and geometry that affected the shrinkage distribution which cannot be detected by traditional LVDTs [35,37]. Therefore, the technique can be used as an optional method to reflect the displacement and deformation of the specimens.

### 3.3. Stress–Strain Relationship

#### 3.3.1. Compressive Stress–Strain Relationship

**Figure 12:**
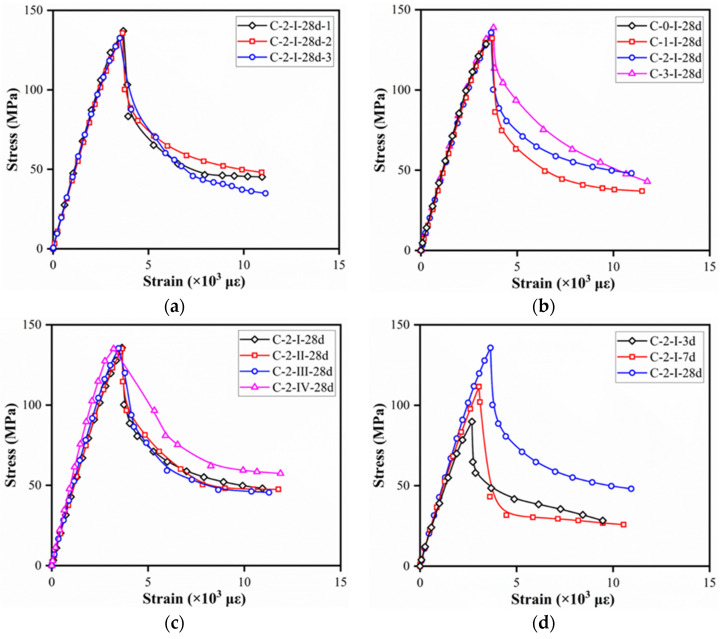
Typical compressive stress–strain curves (ϕ100 × 200 mm^2^ cylinders): (**a**) control specimens (C-2-I-28d); (**b**) fiber volume contents; (**c**) fiber types; (**d**) curing ages.

Figure 12 illustrates the compressive stress–strain curves of reference specimens with different fiber volume contents, fiber types, and curing ages. At least three specimens from every set of specimens were tested and specimen with the middle value was presented. The strain was attained by dividing the average of the LVDT deformations by the LDVT gauge length, while stress was obtained by dividing the load by the cross-sectional area of reference specimens.

As shown in Figure 12a, specimens C-2-I-28d presented an almost linear ascending portion of the stress–strain compressive curves. The load capacity after peak stress dropped at high rates due to the appearance of macrocracks. Afterward, the rate of load decreasing decreased with the bridging effect of fibers and the redistribution of stress. For specimens with different fiber volume contents, a slight increase in the ultimate compressive strength before the cracks fully developed was obtained. The post-peak stage was obvious in specimens with fibers due to the energy absorption and stress redistribution provided by the fiber bridging effect. In contrast, a sudden load drop was observed in specimens without fiber, which related to the energy released with macrocracks developed rapidly after peak stress. In addition, higher fiber content would lead to higher residual strength. For a given fiber content, specimens with hooked-end fibers tended to exhibit insignificant differences in stress–strain curves, while those with straight fiber showed lower peak strain (strain corresponding to peak stress) but higher residual strength. This may be related to larger slipping occurred between the straight fiber and matrix material compared to hooked-end fibers, which could also be found in a single fiber pull out test conducted by Wille et al. [38]. Obvious difference was observed among specimens with various curing ages, as shown in Figure 12d. Larger post-peak load loss was measured for specimens tested at 7 days, which was also found by Hassan et al. [34]. The possible reason was that higher compressive strengths occurred in the matrix at 7 days, while the fiber bridging effect was not strong enough. Therefore, bonding between fiber and matrix could not ensure the redistribution of stress after peak stress.

**Figure 13:**
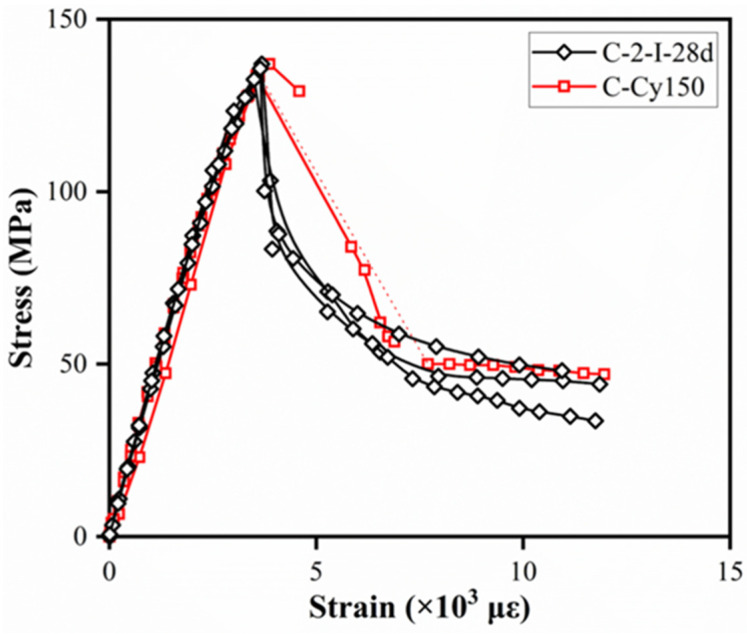
Compressive stress–strain curves for cylinders with different dimensions.

Additionally, ϕ150 × 300 mm^2^ cylinder specimens were tested to investigate the geometric dimension effect on compressive stress–strain curves. Comparison of stress–strain curves between control specimens (C-2-I-28d) and ϕ150 × 300 mm^2^ cylinder specimens (C-Cy150) are given in Figure 13. Several LVTDs of ϕ150 × 300 mm^2^ cylinder specimens were detached from the frame because of the sudden energy release at peak stress. Cylinders with different dimensions presented a similar compressive behavior, especially in the pre-peak stage. However, the descending portion of larger specimens was as steep as the ascending potion. Higher residual strength occurred in ϕ150 × 300 mm^2^ cylinders, which possibly attributable to the geometric dimension effect on the post-peak stage of compressive stress–strain curves.

#### 3.3.2. Tensile Stress–Strain Curve

**Figure 14:**
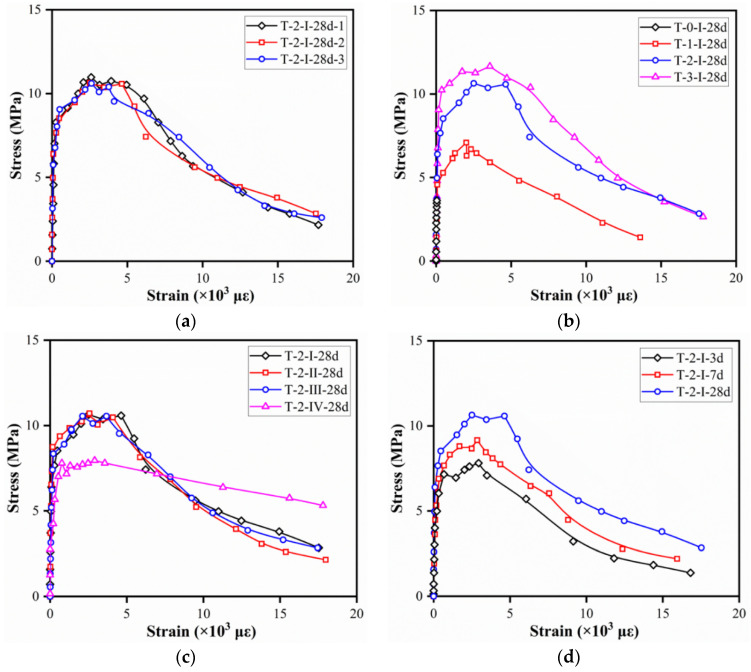
Typical tensile stress–strain curves (dog-bone-shaped specimens): (**a**) control specimens (T-2-I-28d); (**b**) fiber volume contents; (**c**) steel fiber types; (**d**) curing ages.

The stress–strain curves measured by the dog-bone-shaped specimens are depicted in Figure 14. The strain was obtained by dividing the average of the LVDTs extension within the 80 mm test area in the span, while stress was obtained by dividing the machine load by the cross-sectional area of the narrow section of the specimens.

The stress–strain curves of specimens T-2-I-28d were composed of four phases, i.e., the elastic phase, strain–harden phase, low strain–hardening phase, and the strain–soften phase, as presented in Figure 14a. For the specimens in the tensile test, the applied load also increased linearly at the elastic phase. At the strain–harden and low strain–hardening phases, yield strengthening and microcracks development were observed. First and second peak stresses were found at the end of strain–harden phases and low strain–hardening phases, respectively. As the low strain–harden phase ended, stress decreased with the increase in the displacement due to the gradual failure of the fiber bridging effect. The effect of steel fiber volume contents on the tensile stress–strain curves of the specimens is shown in Figure 14b. For specimens without fiber, microcracks developed rapidly after the elastic phase and led to the failure of specimens. Thus, the results of the T-0-II-28d reflected the tensile strength of the matrix in UHPC. In addition, the initial cracking and first peak stress increased with the fiber content and curing age. However, the enhancement on tensile performance was insignificant when fiber content exceeded 2%. As can be seen in Figure 14c, specimens with straight fiber showed a rapid increase in strain with stress decreasing in the descending portion. The differences of the descending portion in Figure 14c indicated better tensile performance could be achieved owing to the stronger bonding between hooked-end fibers and matrix. As for the specimens with different curing ages, strain amplitude at the elastic phase increased when the specimens were loaded at an early age, which was related to the low material stiffness at an early age.

**Figure 15:**
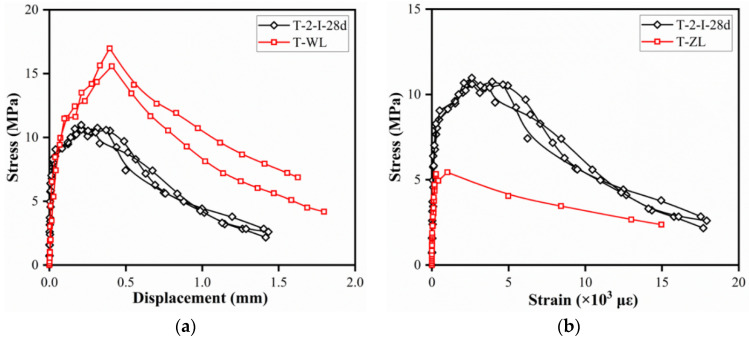
Tensile stress–displacement/strain curves for specimens with different geometries: (**a**) direct tensile and four-point bending tests; (**b**) direct and axial tensile tests.

As shown in Figure 15a, tensile stress–displacement curves in the four-point bending test consisted of elastic phase, hardening phase, and softening phase. The deflection of prism specimens increased linearly until the formation of cracks. As the applied load rose, a linear inflection point was found at the beginning of the deflection hardening phase. However, high peak stress prior to the tension softening phase revealed a high energy ability occurred in prism specimens [16,39]. As for the axial tensile test, specimens failed rapidly after the stress exceeded the ultimate tensile stress of the matrix (3.64 MPa). It was attributed to the peak strain at the single crack location being greater than the ultimate limit strain of the matrix by Hassan et al. [34].

### 3.4. Mechanical Properties of Specimens

#### 3.4.1. Elastic Modulus and Poisson’s Ratio

**Figure 16:**
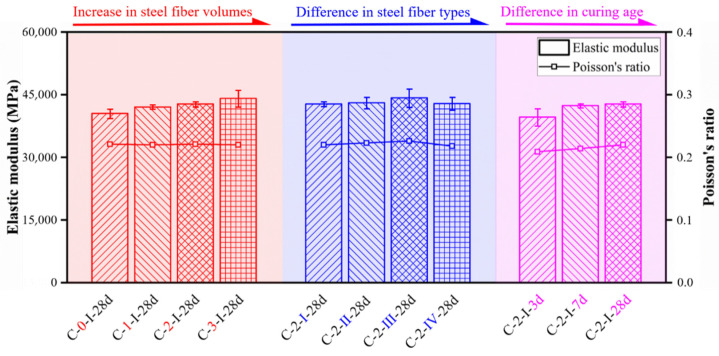
Elastic modulus and Poisson’s ratio for ϕ100 × 200 mm^2^ cylinders.

The method recommended by ASTM C496 [29] was adopted to obtain the elastic modulus and Poisson’s ratio of UHPC. The mean values of experimental results in compression are shown in Table 4. The effect of fiber volume content, fiber type, and curing age on the elastic modulus and Poisson’s ratio can also be found in Figure 16.

For the UHPC with different fiber volume contents, increasing fiber content could slightly improve the initial stiffness. Compared to the specimen without fiber (C-0-I-28, 40518 MPa), specimens with fiber volume contents of 1%, 2%, and 3% exhibited an increase in elastic modulus of 3.81%, 5.58%, and 8.86%, respectively. A similar conclusion was found by Yang et al. [40]. However, the difference in elastic modulus among specimens with different types of fibers was less than 4% for a given fiber content (2%). Moreover, Poisson’s ratio was insignificant changed as fiber content or type varied, which was around 0.220. In addition, the average elastic moduli were 39,638, 42,378, and 42,780 MPa for specimens cured at the age of 3, 7, and 28 days, respectively. The corresponding Poisson’s ratios were 0.209, 0.214, and 0.221, respectively. Therefore, longer curing age did produce a slight improvement (about 5%) in the elastic modulus and Poisson’s ratio.

#### 3.4.2. Compressive Strength and Corresponding Stress

**Figure 17:**
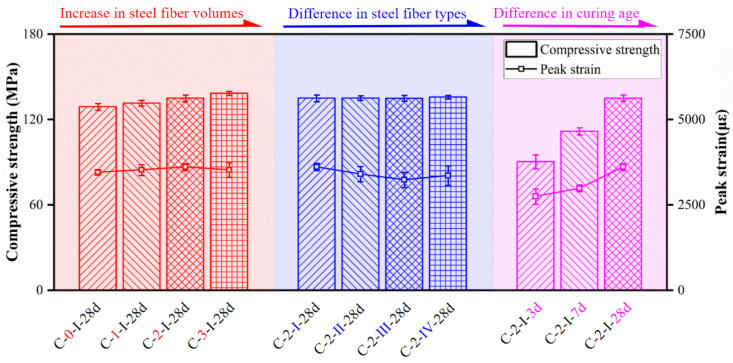
Compressive strength and corresponding peak strain.

Figure 17 depicts the effect of fiber volume content, fiber type, and curing age on the measured ultimate compressive strength and corresponding strain of reference compressive specimens. The steel fiber volume content and type showed a very limited effect on the compressive performance of the specimens. Peak stress increased with the increase in steel fiber volume content. The peak stress of UHPCs using steel fibers with volume contents of 1%, 2%, and 3% were increased by 1.90%, 4.70%, and 7.32%, respectively, as compared to specimens C-0-I-28d. However, specimens with different fiber contents showed an insignificant difference in peak strain, which was mainly attributed to the negative effect of fiber volume content on flowability [40]. Moreover, specimens with different fiber types showed little variation both in compressive strength and peak strain. Peak strain of specimens with different fiber contents and types were measured to be about 3500 με. In addition, specimens exhibited massive differences due to the different matrix strengths at different ages. The average strengths measured at 3 and 7 days were 66.97% and 82.75% of those at 28 days, respectively. Furthermore, peak strain demonstrated a tendency to increase with curing age as well. Peak strains of specimens testing at 3 and 7 days were only 2751 and 2986 με, respectively, far less than 3612 με (C-2-I-28d).

Additionally, the results of specimens with different geometric dimensions are presented in Table 5. It seemed that the strengths were almost independent of the dimensions of the cylinders. However, higher strengths were measured on smaller cubes. The compressive strengths of cube specimens with side lengths of 70, 100, and 150 mm were 146.85, 135.01, and 123.53 MPa, respectively. In the study conducted by Fládr and Bílý [13], mixture with compressive strength of about 130 MPa (100 × 100 × 100 mm^3^ Cube) showed a similar tendency.

**Figure 18:**
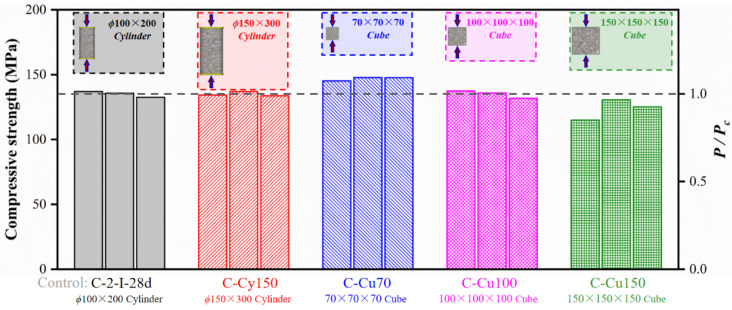
Compressive strength of specimens with different geometric dimensions.

The geometric dimension effect on compressive strength of UHPC is depicted in Figure 18. Generally, the plain concrete cylinder specimen compressive strength could be transferred to the cube strength by conversion factors. Moreover, the factor became closer to 1.00 as the concrete strength increased [41]. Coincidentally, the measured strength of 100 mm cubes and two sizes of cylinders were almost the same. One possible reason was that the incorporation of fiber resulted in a decrease in the coefficient of variation of the compressive strength [31]. However, conversion factors to transform the strength measured on 75 and 150 mm cubes to the strength of cylinders were 1.09 and 0.92, respectively. Compressive strengths relative to 100 mm cube measured on cylinder and cube with different dimensions are summarized in Table 5.

#### 3.4.3. Tensile Strength and Corresponding Stress

**Figure 19:**
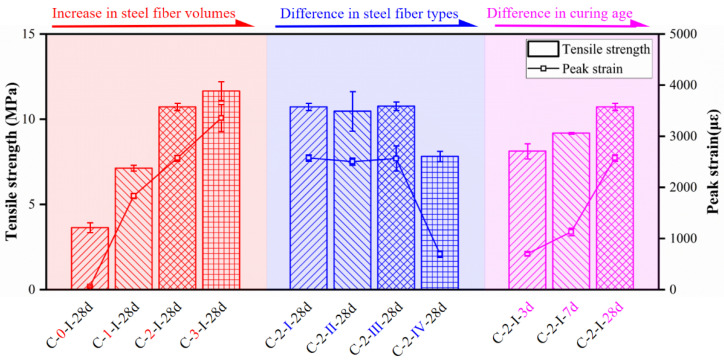
Tensile strength and corresponding peak strain.

The mean values of tensile strength and peak train measured by dog-bone-shaped specimens are presented in Table 6 and Figure 19. The results revealed that the ultimate tensile strength and corresponding strain increased as fiber content and curing age increased. For specimens with different fiber contents, specimens T-3-I-28d exhibited the highest mean peak stress and corresponding strain, which increased by 220% and 5688%, respectively, compared to those without fibers. For the notched prism specimens axial tensile test, the increase in fiber volume content elevated the ultimate tensile capacity as well [12]. In addition, the mean peak stress and peak strain of specimens T-2-I-28d were increased by 32% and 267%, respectively, compared to those tested at 3 days. However, specimens with straight fibers showed lower strengths and strains than those with hooked-end fibers with the same fiber content.

**Figure 20:**
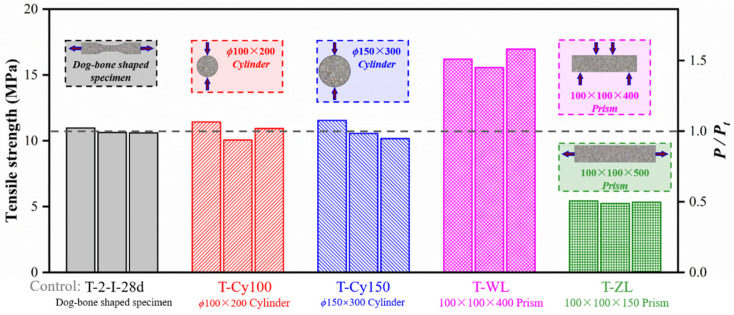
Comparison of tensile strength from different types of specimens.

Tensile tests on a variety of specimens with different geometric dimensions were also conducted in this study, as presented in Table 7 and Figure 20. Compared with previous studies [13,24], more geometric dimensions of tensile specimens were considered in the study and more conversion factors were proposed. ϕ100 × 200 mm^2^ cylinders and ϕ150 × 300 mm^2^ cylinders showed similar splitting tensile strengths as compared with dog-bone-shaped specimens (T-2-I-28d). However, larger peak stress was obtained in the four-point bending test owing to more fibers being involved in the bridging effect as cracks developed. A mean flexural value of 16.24 MPa was measured, which was 1.51 times the mean strength of the control dog-bone-shaped specimens. Peak stress obtained from the axial tensile test (T-ZL) was half the result of the specimens T-2-I-28d, which might be explained by the larger dimension leading to random distribution of fibers. In addition, Wille et al. suggested that the small height and width of the specimen mold in comparison to a fiber length would lead to fiber alignment in the direction of the applied tensile [16]. Thus, fibers in the dog-bone specimen contributed more tension than those in prism specimens (T-ZL).

## 4. Constitutive Law

As a basic index for the design and analysis of concrete structures, the definition of constitutive law is particularly important. The constitutive laws played an important role in finite element analysis as well. Constitutive laws could be used to determine whether the mechanical properties of UHPC meet the code requirements. In addition, the constitutive relation could also apply to compare the mechanical properties of non-proprietary UHPC with those of proprietary UHPC. However, to the best of our knowledge, the constitutive laws proposed by codes and previous studies did not fully consider the role of steel fibers in UHPC. AFGC recommendation [23] took the fiber orientation into account, while constitutive laws in other studies were only applicable to a given fiber type [12,24,25,26]. The proposal of UHPC constitutive laws that fully consider the role of fibers is of great importance. Herein, both compressive and tensile constitutive laws took fiber geometry, fiber length and fiber volume content as factors were developed.

### 4.1. Compressive Constitutive Law

One of the most widely accepted models for generating the complete compressive stress–strain curve of UHPC is presented by Equation (1), which was proposed by Yang et al. [20] based on the FIB Model Code [42].

(1) $$\sigma_{c}=\left\{ \begin{array}{l} f_{c}^{\prime }\frac{n\xi -\xi^{2}}{1+\left(n-2\right)\xi }\\ f_{c}^{\prime }\frac{\xi }{2{\left(\xi -1\right)}^{2}+\xi } \end{array}\right.$$

where *f_c_*′ = compressive strength of UHPC (MPa); *ξ* = strain ratio; *ξ* = *ε*/*ε*_0_; *ε*_0_ = peak strain (με); *n* = *E_c_/E_sec_*; *E_c_* = initial elastic modulus (MPa); *E_sec_* = cenotes secant modulus at *f_c_*′ (MPa).

Another classical constitutive law with an exponential form was proposed by Nielsen [19], as shown in Equation (2).

(2) $$\frac{\sigma }{f_{c}^{\prime }}=\frac{2.5\frac{\varepsilon }{\varepsilon_{0}}}{1.5+{\left(\frac{\varepsilon }{\varepsilon_{0}}\right)}^{2}}$$

In addition, various polynomial representations obtained by the linear analysis were also developed. The polynomial expression proposed by Li et al. was listed [22], as illustrated in Equations (3) and (4).

(3) $$y_{1}=\left\{ \begin{array}{l} 1.55x_{1}-1.20x_{1}^{4}+0.65x_{1}^{5}\\ \frac{x_{1}}{6{\left(x_{1}-1\right)}^{2}+x_{1}} \end{array}\right.$$

(4) $$x_{1}=\frac{\varepsilon }{\varepsilon_{0}},y_{1}=\frac{\sigma }{\sigma_{0}}$$

**Figure 21:**
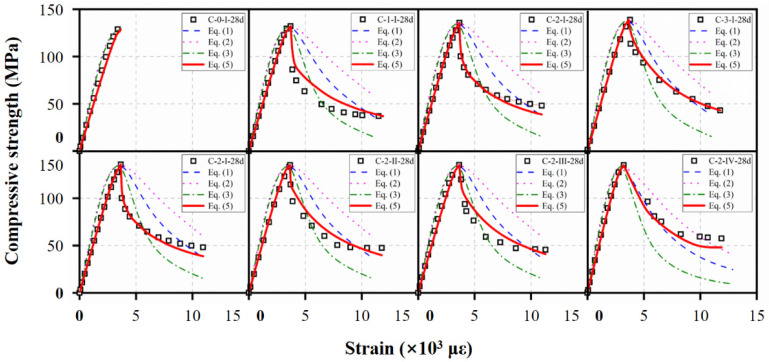
Comparison of different compressive constitutive laws.

Comparison among compressive constitutive laws mentioned above and the test data are shown in Figure 21. The models were capable of predicting the stress–strain behavior well in the ascending portion of UHPC. However, the descending part cannot be well described due to the sudden drop after peak stress. As discussed previously, the descending portion was influenced by the fiber contents and types, which were not considered in the equations above. Since comparison of the experimental results revealed the original form of pre-peak ranges in Equation (1) could accurately describe the ascending portions, the same model was adopted in this study. For the descending portions, two correction coefficients, *α*, and *μ*, were introduced to reflect the effect of fibers. The expression forz compressive stress–strain curve of UHPC was proposed as Equations (5) and (6).

(5) $$\sigma_{c}=\left\{ \begin{array}{l} f_{c}^{\prime }\frac{n\xi -\xi^{2}}{1+\left(n-2\right)\xi }\\ f_{c}^{\prime }\frac{\alpha \xi }{\xi^{2}+\mu \xi } \end{array}\right.$$

(6) μ=−0.01×l×v+0.48

where *α* = a coefficient related to steel fiber types (*α* = 1.2 for straight fiber; *α* = 1.0 for hooked-end fiber); *μ* = a coefficient related to fiber length and volume content; *l* = fiber length (mm); *v* = fiber volume content.

The prediction results of reference compressive specimens obtained from Equation (5) are presented in Figure 21. As discussed earlier, Equation (5) could describe accurately the pre-peak ranges of the curves. In addition, the descending portions were well predicted by Equation (5) for UHPC with different fiber contents and types.

### 4.2. Tensile Constitutive Law

Several constitutive laws for predicting the tensile stress–strain relationship of UHPC were reported in the study. One of the most widely used models was the three-stage expression proposed by Zhang et al. [21], in which the linear elastic stage and the strain–hardening segment were expressed in the form of the stress–strain curve. The expression is presented by Equation (7).

(7) $$\sigma_{t}=\left\{ \begin{array}{ll} \frac{f_{ct}}{\varepsilon_{ca}}\varepsilon & 0\leq \varepsilon \leq \varepsilon_{ca}\\ f_{ct} & \varepsilon_{ca}\leq \varepsilon \leq \varepsilon_{pc}\\ f_{ct}\frac{1}{{\left(1+w/w_{p}\right)}^{p}} & 0<w \end{array}\right.$$

where *f_ct_* = tensile strength of UHPC (MPa); *ε_ca_* = strain at beginning of strain–harden after peak stress (*με*); *ε_pc_* = strain at the end of the strain–hardening before the descending portion (με); *w_p_* = crack width at 2*^−p^f_ct_* (mm); *p* = the experimental fitting parameter, which is 0.95 [43].

Another model with good acceptance was a multiple linear expression developed by Liao et al. [23], as shown in Equation (8).

(8) $$\sigma_{t}=\left\{ \begin{array}{ll} \frac{\varepsilon }{\varepsilon_{cc}}\sigma_{cc} & 0\leq \varepsilon \leq \varepsilon_{cc}\\ \sigma_{cc}+\frac{\varepsilon -\varepsilon_{cc}}{\varepsilon_{pc}-\varepsilon_{cc}}\left(\sigma_{pc}-\sigma_{cc}\right) & \varepsilon_{cc}\leq \varepsilon \leq \varepsilon_{pc}\\ \left(1-\frac{\varepsilon -\varepsilon_{pc}}{\varepsilon_{u}-\varepsilon_{pc}}\right)\sigma_{pc} & \varepsilon >\varepsilon_{pc} \end{array}\right.$$

where *σ_cc_* = stress corresponding to the beginning of strain–harden (*με*); *ε_cc_* = strain at the beginning of strain–harden (*με*); *ε_pc_* = strain at the end of strain–harden (*με*); *ε_u_* = ultimate strain.

Additionally, Li et al. [22] also proposed a tensile constitutive law applicable to RPC by linear fitting, as shown in Equations (9) and (10).

(9) $$y_{2}=\left\{ \begin{array}{l} 1.17x_{2}-0.65x_{2}^{2}-0.83x_{2}^{3}\\ \frac{x_{2}}{5.5{\left(x_{2}-1\right)}^{2.2}+x_{2}} \end{array}\right.$$

(10) $$x_{2}=\frac{\varepsilon }{\varepsilon_{t}},y_{1}=\frac{\sigma }{\sigma_{t}}$$

where *σ_t_* = peak stress; *ε_t_* = strain at peak stress.

**Figure 22:**
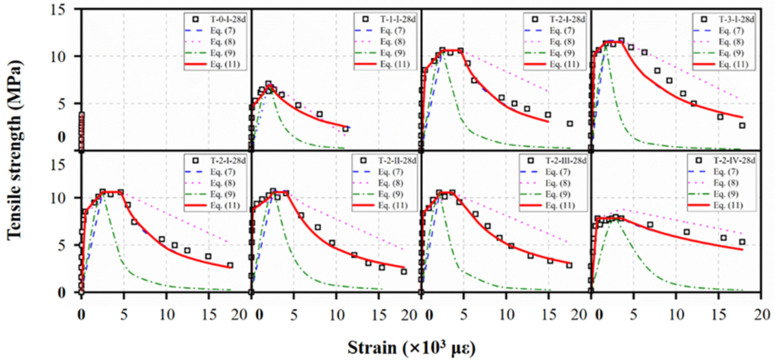
Comparison of different tensile constitutive laws.

The prediction of the above models was compared with the tensile stress–strain curves, as shown in Figure 22. Equation (7) showed large deviations in the pre-peak behavior, while presenting accurate prediction for the strain–softening portion. However, Equation (8) described the elastic phase accurately, while it exhibited insufficient prediction for the descending portion. Equation (9), which considered the peak strain but ignored the strain–harden stage, showed poor prediction for complete curves.

It was already known that the initial and strain–softening stages were well predicted Equations (7) and (8), respectively. On the basis of models mentioned above and the properties of tensile stress–strain curves, a four-stage expression that considered the elastic stage, the strain–harden range, the low strain–hardening range after first peak stress, and the strain–soften range after second peak stress was proposed, as shown by Equation (11). The mean value of the first and second peak stresses was used as the average stress in the low strain–harden range after the first peak stress.

(11) $$\sigma_{t}=\left\{ \begin{array}{ll} \frac{\varepsilon }{\varepsilon_{cc}}\sigma_{cc} & 0\leq \varepsilon \leq \varepsilon_{cc}\\ \frac{\varepsilon -\varepsilon_{cc}}{\varepsilon_{u1}-\varepsilon_{cc}}\left(f_{ct}-\sigma_{cc}\right)+\sigma_{cc} & \varepsilon_{cc}\leq \varepsilon \leq \varepsilon_{u1}\\ f_{ct} & \varepsilon_{u1}\leq \varepsilon \leq \varepsilon_{u2}\\ f_{ct}\frac{1}{{\left(1+w/w_{p}\right)}^{p}} & 0<w_{p} \end{array}\right.$$

where *ε*_*u*1_ = strain at first peak stress (*με*); *ε*_*u*2_ = strain at second peak stress (*με*); *f_ct_* = average of the two peak stresses (MPa).

The prediction results of reference tensile specimens obtained from Equation (11) as compared in Figure 22 with experimental results showed a good agreement. Due to the strain–hardening and low strain–hardening behaviors, a four-stage model should be considered when evaluating the tensile stress–strain relationship of UHPC.

## 5. Conclusions

In this study, compressive and tensile tests were conducted to explore the mechanical properties of UHPC with different steel fibers and specimen geometric dimensions. The effects of fiber volume content, fiber type, curing age and geometric dimension were investigated. Based on the experimental results, the following conclusions can be drawn:

1. The increase in steel fiber volume contents led to the reduction in flowability of fresh UHPC. On the contrary, unit weight increased with the incorporation of steel fibers. However, no noticeable variations occurred in the workability of UHPC with different fiber types;
2. As for compressive properties, compressive stress and elastic modulus were improved with increasing fiber volume content and curing age, regardless of the fiber types. Poisson’s ratio was insignificantly influenced by fiber volume content and type, and increased slightly with curing age;
3. The tensile performance of UHPC was substantially influenced by the increase in the fiber volume content and curing age. In addition, specimens with hooked-end fibers obtained better tensile properties than those with straight fibers.
4. Specimens in tensile tests exhibited more a significant geometric dimension effect than those in compressive tests, which was attributed to the different loading procedures of the tensile tests and the random distribution of fibers in the tensile specimens. The compressive strength of the cylinder was closed to 100 mm cube compressive strength. Conversion factors were proposed for the transformation of compressive strengths measured on a 70 or 150 mm cube and cylinder to the strength of 100 mm cube. The results measured from the direct tensile test and the splitting tensile test showed insignificant deviations. However, the mean tensile strength of prisms specimens in the axial tensile test and four-point bending test was about 0.50 and 1.51 times of those obtained from dog-bone-shaped specimens, respectively;
5. The compressive stress–strain curves of UHPC with different parameters exhibited a sudden load drop after peak strength, while tensile stress–strain curves were divided into four phases. Based on the existing constitutive laws and comparison with results of those laws, the compressive constitutive law taking into account the effect of fiber content and fiber type was proposed. In addition, a four-stage tensile constitutive law was suggested to describe the tensile behavior of UHPC.

## Figures and Tables

**Table 1:** Properties of steel fibers.

Type	Diameter *d_f_* (mm)	Length *l_f_* (mm)	Aspect Ratio (*l_f_*/*d_f_*)	Tensile Strength (MPa)	Elastic Modulus (GPa)	Fiber Type
Type I	0.22	13	59.09	2600	200	Hooked-end
Type II	0.22	16	72.73	2500	200	Hooked-end
Type III	0.25	16	64.00	2600	200	Hooked-end
Type IV	0.20	13	65.00	2800	200	Straight

**Table 2:** Mixture proportions of UHPC.

No.	UHPC Mixes Design (kg/m^3^)							Fiber Volume Content (%)	Fiber Type	Slump Flow (mm)	Unit Weight (kg/m^3^)
	Cement	Silica Fume	Silica Sand	Nano-CaCO_3_	Water	Water Reducer	Steel Fiber				
I	829	216	1079	35	194	29	0	0	-	225	2294
II							78	1	Type I	222	2392
III							156	2	Type I	217	2429
IV							234	3	Type I	212	2498
V							156	2	Type II	228	2441
VI							156	2	Type III	232	2456
VII							156	2	Type IV	220	2441

**Table 3:** Details of concrete material test specimens.

Type	Specimen	Specimen Geometry	Fiber Volume Content	Fiber Type	Age
Compression	C-0-I-28d	ϕ100 × 200 mm^2^ cylinders (Reference compressive specimens)	0	Type I	28 d
	C-1-I-28d		1%	Type I	28 d
	C-2-I-28d		2%	Type I	28 d
	C-3-I-28d		3%	Type I	28 d
	C-2-II-28d		2%	Type II	28 d
	C-2-III-28d		2%	Type III	28 d
	C-2-IV-28d		2%	Type IV	28 d
	C-2-I-3d		2%	Type I	3 d
	C-2-I-7d		2%	Type I	7 d
	C-Cy150	ϕ150 × 300 mm^2^ cylinders	2%	Type I	28 d
	C-Cu70	70 × 70 × 70 mm^3^ cubes	2%	Type I	28 d
	C-Cu100	100 × 100 × 100 mm^3^ cubes	2%	Type I	28 d
	C-Cu150	150 × 150 × 150 mm^3^ cubes	2%	Type I	28 d
Tension	T-0-I-28d	dog-bone shaped specimens (Reference tensile specimens)	0	Type I	28 d
	T-1-I-28d		1%	Type I	28 d
	T-2-I-28d		2%	Type I	28 d
	T-3-I-28d		3%	Type I	28 d
	T-2-II-28d		2%	Type II	28 d
	T-2-III-28d		2%	Type III	28 d
	T-2-IV-28d		2%	Type IV	28 d
	T-2-I-3d		2%	Type I	3 d
	T-2-I-7d		2%	Type I	7 d
	T-Cy100	ϕ100 × 200 mm^2^ cylinders	2%	Type I	28 d
	T-Cy150	ϕ150 × 300 mm^2^ cylinders	2%	Type I	28 d
	T-WL	100 × 100 × 400 mm^3^ prisms	2%	Type I	28 d
	T-ZL	100 × 100 × 500 mm^3^ prisms	2%	Type I	28 d

**Table 4:** Experimental results for compressive tests (ϕ100 × 200 mm^2^ cylinders).

Specimen	Compressive Strength (MPa)	Peak Stain (με)	Elastic Modulus (MPa)	Poisson’s Ratio
C-0-I-28d	129.01	3455	40,518	0.221
C-1-I-28d	131.46	3527	42,061	0.220
C-2-I-28d	135.07	3612	42,780	0.221
C-3-I-28d	138.45	3525	44,107	0.220
C-2-II-28d	135.09	3403	43,051	0.223
C-2-III-28d	134.86	3235	44,241	0.226
C-2-IV-28d	135.74	3359	42,877	0.218
C-2-I-3d	90.46	2751	39,638	0.209
C-2-I-7d	111.77	2986	42,378	0.214

**Table 5:** Comparison of compressive strength of specimens with different geometric dimensions.

ϕ150 × 300 mm^2^ Cylinders (MPa)		70 × 70 × 70 mm^3^ Cubes (MPa)		100 × 100 × 100 mm^3^ Cubes (MPa)		150 × 150 × 150 mm^3^ Cubes (MPa)	
Mean (*P_cy_*_150_)	134.99	Mean (*P_cu_*_70_)	146.85	Mean (*P_cu_*_100_)	135.01	Mean (*P_cu_*_150_)	123.53
*P_cy_*_150_*/P_c_* ^1^	1.00	*P_cu_*_70_ */P_c_*	1.09	*P_cu_*_100_ */P_c_*	1.00	*P_cu_*_150_ */P_c_*	0.91

^1^ *Pc* = Mean compressive strength of specimens C-2-II-28d.

**Table 6:** Experimental results for the tensile test (dog-bone-shaped specimens).

Specimen	Tensile Strength (MPa)	Strength Error ^1^ (%)	Peak Stain (με)	Strain Error ^2^ (%)
T-0-I-28d	3.64	−66	58	−98
T-1-I-28d	7.13	−34	1834	−29
T-2-I-28d	10.73	0	2576	0
T-3-I-28d	11.66	9	3357	30
T-2-II-28d	10.47	−2	2503	−3
T-2-III-28d	10.77	0	2564	0
T-2-IV-28d	7.82	−27	698	−73
T-2-I-3d	8.13	−24	701	−73
T-2-I-7d	9.19	−15	1130	−56

^1^ Strength error = (specimen tensile strength—T-2-I-28d tensile strength)/T-2-I-28d tensile strength; ^2^ Strain error = (specimen peak strain—T-2-I-28d peak strain)/T-2-I-28d peak strain.

**Table 7:** Comparison of tensile strength of specimens with different geometric dimensions.

ϕ100 × 200 mm^2^ Cylinders (MPa)		ϕ150 × 300 mm^2^ Cylinders (MPa)		100 × 100 × 400 mm^3^ Prisms (MPa)		100 × 100 × 500 mm^3^ Prisms (MPa)	
Mean (*P_sp_*_100_)	10.81	Mean (*P_sp_*_150_)	10.75	Mean (*P_WL_*)	16.24	Mean (*P_ZL_*)	5.33
*P_sp_*_100_*/P_t_* ^1^	1.01	*P_sp_*_150_ */P_t_*	1.00	*P_WL_/P_t_*	1.51	*P_ZL_/P_t_*	0.50

^1^ *P_t_* = Mean tensile strength of specimens T-2-II-28d.

## Data Availability

Not applicable.
